# Digital tools as promoters for person-centered care practices in chronic care? Healthcare professionals’ experiences from rheumatology care

**DOI:** 10.1186/s12913-020-05945-5

**Published:** 2020-12-01

**Authors:** Emma Granström, Carolina Wannheden, Mats Brommels, Helena Hvitfeldt, Monica E. Nyström

**Affiliations:** 1 Department of Learning, Informatics, Management and Ethics, Medical Management Centre, Karolinska Institutet, SE-17177 Stockholm, Sweden; 2 Norrtälje Hospital, FoUU, SE-76129 Norrtälje, Sweden; 3 Department of Epidemiology and Global health, Umeå University, SE-90187 Umeå, Sweden

**Keywords:** Patient-centered care, Person-centered care, Digital tools, Chronic care, Improvement

## Abstract

**Background:**

Person-centered care (PCC) emphasize the importance of supporting individuals’ involvement in care provided and self-care. PCC has become more important in chronic care as the number of people living with chronic conditions is increasing due to the demographic changes. Digital tools have potential to support interaction between patients and healthcare providers, but empirical examples of how to achieve PCC in chronic care and the role of digital tools in this process is limited. The aim of this study was to investigate strategies to achieve PCC used by the healthcare professionals at an outpatient Rheumatology clinic (RC), the strategies’ relation to digital tools, and the perceived impact of the strategies on healthcare professionals and patients.

**Methods:**

A single case study design was used. The qualitative data consisted of 14 semi-structured interviews and staff meeting minutes, covering the time period 2017–2019. The data were analyzed using conventional content analysis, complemented with document analyses.

**Results:**

Ten strategies on two levels to operationalize PCC, and three categories of perceived impact were identified. On the individual patient level strategies involved several digital tools focusing on flexible access to care, mutual information sharing and the distribution of initiatives, tasks, and responsibilities from provider to patients. On the unit level, strategies concerned involving patient representatives and individual patients in development of digital services and work practices. The roles of both professionals and patients were affected and the importance of behavioral and cultural change became clear.

**Conclusions:**

By providing an empirical example from chronic care the study contributes to the knowledge on strategies for achieving PCC, how digital tools and work practices interact, and how they can affect healthcare staff, patients and the unit. A conclusion is that the use of the digital tools, spanning over different dimensions of engagement, facilitated the healthcare professionals’ interaction with patients and the patients’ involvement in their own care. Digital tools complemented, rather than replaced, care practices.

## Background

Healthcare systems in many countries face challenges in enhancing quality of care while adjusting to demographic changes with ageing populations. A particularly relevant area is chronic care as chronic conditions increase with age. It is anticipated that a higher proportion of the population will be living with chronic and life-style related conditions in the future [1] and the importance of improving the management of long-term and chronic conditions will increase as the number of patients grow [2].

A way to improve the management of chronic care is to increase patients’ involvement in their own health (e.g. preventive measures) and in decisions regarding the treatment of their conditions [3, 4] and such practices are associated with positive outcomes for patients with chronic conditions [5–7]. Most people with chronic conditions spend most of their time in self-care, i.e. taking care of themselves or adopting their behaviors to prevent illness [8]. This requires self-management, i.e. the ability to manage symptoms, treatment, physical and psychosocial consequences and lifestyle changes inherent when living with a chronic condition [9]. While only a fraction of a patient’s time is spent in healthcare settings, healthcare often lacks processes and tools to support individuals’ self-care. Therefore, new types of partnerships between patients and healthcare professionals have been called for [10, 11].

Partnerships between patients and healthcare professionals have been described in many ways in the literature. For example, there are numerous conceptual frameworks of patient-centered and person-centered care [12–15]. *Patient-centered care* involves the planning of treatment and care together and in shared understanding with the patient, and a practitioner-patient collaboration based on partnership [16]. Over time, the term *person-centered* has been used more and more [17] as it broadens and extends patient-centered care by considering the whole life of the person beyond the clinical or medical condition [18], including the importance of knowing the person and their context in order to engage as active partners [19].

According to a recent synthesis of reviews [18], there are several similarities between patient-centered care and person-centered care as they both include elements of empathy, respect, engagement, relationship, communication, shared-decision making, holistic focus, individualized focus and coordinated care. What differs between the two concepts is mainly the goal – patient-centered care aims for a functional life for the patient while person-centered care aims for a meaningful life for the person [18]. In chronic care, differences have also been described in terms of continuity of interactions between the individual and their healthcare providers, and how preferences, needs, and values may evolve over time and influence that care [20]. Regardless of the differences between the two concepts, patient and person-centered care coexist in clinical practice. There is also an overlap between the two concepts as physical functions influence a person’s life-situation and vice versa. Therefore, we address both concepts in this study, not to exclude any important information. We will use the term *PCC practices* to address both patient-centred and person-centred practices in order to capture both improvement of patients’ function and life situation.

Other terms are also used to emphasize the collaboration between patients and healthcare professionals at different levels of the healthcare system, including *patient participation* [21], *co-production of care* [22], *patient engagement* [23], and *co-care* [24]. The definition of co-care particularly highlights the importance of using information and communication technology (ICT) to enhance the interaction and collaboration between patients and healthcare professionals. The use of ICT can increase the patients’ access to timely, sufficient and appropriate health information that can aid decisions about their own care and desired level of engagement, and thereby support self-care and PCC [25–28]. ICT is seen as an important enabler of patient-provider partnerships [25] that may accelerate the transition towards PCC [29]. This is a recent development and the understanding of ICT as a support in PCC is at its early stages [25]. More knowledge has been called for on the actual use of ICT and how ICT can support the partnership between patients and healthcare professionals [25]. While digital tools are regarded as promising for the facilitation of communication, monitoring of disease, and for educational support, they are not yet fully used in practice [30]. If patients can become more informed, influential and active it is anticipated that this will change the way healthcare professionals work as well as how care is provided [31].

Globally, healthcare systems are moving towards patient- or person-centeredness [32–34], which requires the re-design of services, structures and roles [15]. Empirical studies of PCC often focus on the actual meetings between care provider and patient and less on the organizational, structural or policy levels [35, 36]. There is still limited knowledge about the implementation of PCC [15] and on how to organize for involvement of patients in efforts to improve quality [37]. Thus, we argue that there is a need for comprehensive empirical studies on the transfer from a traditional way of delivering chronic care to the application of PCC practices and the role of ICT in this process.

Swedish healthcare has long been dominated by hospitals while primary healthcare has been less developed [38]. To better respond to patients’ needs, a national health reform has been launched for available, geographically close, continuous and integrated care from the patient’s perspective [39]. Care interventions will increasingly move from hospitals to other care facilities, more accessible to patients. This implies a shift from reactive care in hospitals to proactive care in more differentiated forms of care. The reform also emphasizes PCC and integration of health services, supported by eHealth. In this study, we were granted access to one of the first outcomes of this reform, a newly established outpatient rheumatology clinic (RC). The clinic has an explicitly stated mission to develop new services and digital tools in close collaboration with patients and primary care, and to enhance education, learning and research. This is in line with the recommendations by the Swedish Agency for Health and Care Services Analysis that suggests the involvement of patient representatives at an organizational level to co-create services and care processes together with healthcare professionals [36]. The RC offers an empirical platform to study conditions central to the development of PCC practices and the role of digital tools in this process. Accordingly, our study aimed to investigate the overall strategies to achieve PCC used by the healthcare professionals at an outpatient rheumatology clinic (RC), the strategies’ relation to digital tools used, and the perceived impact of the strategies on healthcare staff and patients.

## Methods

A single case study design was used to investigate work practices and digital tools aiming to enhance PCC and quality improvement in a real-life context [40]. The qualitative data consisted of interviews and documents.

### Empirical setting

Swedish healthcare is organized by self-governing regional health authorities and the rheumatology clinic (RC) is located in one of the larger of the 21 regions in Sweden. The RC was established in spring 2016 and employs 45 healthcare professionals (working part or full-time). Approximately 6000 patients with inflammatory arthritis diseases are admitted to the clinic. Beginning in 2017, three more outpatient specialist clinics have successively moved in to the same premises as the RC. These clinics have in common that they treat patients with chronic conditions, are research intensive, and have undergone transformations technically and in treatment. In all these clinics close interaction with patients is seen as essential for successful results. Together they form an academic specialist organization with a special reimbursement model to allow for innovation and development.

The aim of the RC is to provide care that is accessible and corresponds to patients’ needs. For example, one priority has been to enhance the development and use of digital health tools to improve care services and to support PCC. The public platform for digital interaction is the digital Healthcare Guide 1177, which is the national hub for advice, information, inspiration and e-services for health and healthcare. It contains both general health information and information on individuals, which can be accessed by each patient after being securely logged in. The type of information and functions patients and healthcare staff can see and use depends on what services that are offered at a specific clinic. In the current study, we have defined digital tools as the use of electronic means to deliver health-related information, resources, and services [41] and categorized them according to their practical function as part of the analysis procedure described below. At the on-set of the study the RC offered 11 digital tools for patient use (see Table 2 for more information). According to the clinic, all patients were informed and offered to use available digital tools. The actual use by patients varied between the tools, from a few to numerous patients. The RC had no exact measures of patient utilization, but estimated a steady rise between 2017 and 2019.

### Data collection

#### Participants

Participants in the interviews were recruited using purposive sampling (see Table 1 for description of participants). In a first step the unit manager was asked to propose names of healthcare staff that were firmly established, working a majority of their time at the clinic (≥50% of full time employment), and expected to have experience of establishing PCC and digital tools for patient use at the clinic. An additional criterion was variation in professional roles (nurses, physicians, physiotherapists, occupational therapists and any of those in strategic or coordinating roles). The researchers then contacted all the identified healthcare staff and included those who agreed to participate (10 of 13 staff agreed). The first round of interviews was performed at the clinic in the autumn of 2017, 1 year after the establishment of the RC. The first four interviews in this round were conducted in pairs (EG/MD) with one lead interviewer and one person focusing on the need for follow-up questions depending on the participants’ answers. The remaining six interviews were conducted by one person (MD). Preliminary results were then presented to healthcare staff at the unit during a feedback session allowing for discussion on interpretations and identification of any further changes in work practices or digital tools. The feedback session was applied to achieve trustworthy results [42]. Some changes in practices and tools were identified and a second round of interviews with key participants with insight into these changes (*n* = 4) was performed in spring 2019 by one person (EG). These four individuals had also participated in the first round of interviews. In total the material consists of 14 interviews, lasting 30–60 min.

**Table 1:** Description of participants

First round of interviews 2017 - Professions	***n*** = 10
Research coordinator	1
Unit manager/reg. Nurse	1
Reg. nurse (1 with IT strategist role)	2
Assistant nurse	1
Physician	2
Physiotherapist	1
Occupational therapist	2
**Follow-up interviews 2019 - Professions**	**n = 4**
Research coordinator	1
Unit manager/reg. Nurse	1
IT strategist/reg. Nurse	1
Physician	1

#### Interviews

The interview guides were developed specifically for this study in order to capture work practices and strategies to achieve PCC and the role of digital tools used at the clinic in this process (see supplementary files). The first round of semi-structured interviews in 2017 consisted of the healthcare professional’s experiences of: 1) the new organization’s mission and of the existing and emerging work practices; 2) the digital tools and e-health services being introduced and/or used, aiming to trace the relation of the work practices and digital tools to PCC practices and improvement. In the additional interviews in 2019 healthcare staff were expected to have experience regarding new digital tools and work practices related to the interaction with patients. The questions concerned the healthcare professional’s experiences of: 1) work practices in relation to the digital tools being used at the time; and 2) work practices in relation to interactions with patients (PCC) and improvement of care practices.

#### Documents

The interviews were complemented with documentation of formal patient-provider interaction during patient council meetings at the unit, and consisted of minutes from the patient council meetings held between Sept. 2017 – Sept. 2019 (*n* = 10). The documents were used to triangulate some of the descriptions in the interviews for more credible results [40] regarding the role of the patient council and the topics discussed during the patient council meetings.

### Data analysis

All interviews were audio-recorded and transcribed verbatim. An iterative approach was used in the analysis. Two researchers (EG and MN) carefully read the interviews to get a sense of the material. Conventional content analysis [42] was then applied to derive categories from the data in several steps. In the first step, units of text describing *digital tools, patient interaction and work practices* were highlighted and condensed (EG). In a second step, the condensed units of text were classified (EG) into two main categories based on if they concerned: *1) individual patient level*, or *2) the unit level*. Finally, categories describing work approaches in terms of overarching strategies were derived, together with their relation to digital tools and perceived outcomes (EG and CW). The entire analysis process was conducted by the first author (EG) and repeatedly processed and discussed with the last (MN) and the second author (CW).

## Results

The results are presented in three main sections reflecting ten strategies on two levels that the RC employed to operationalize PCC and one section describing the perceived impact of these strategies.

### Strategies at the individual patient level for achieving PCC and improving care

Five strategies at the individual patient level were identified, all involving one or more digital tool for interaction and collaboration between patients and healthcare professionals. Table 2 presents a summary of the digital tools used at the RC and a description of procedures related to their use.

**Table 2:** Digital tools used at the rheumatology clinic and procedures related to their use

#	Digital tools (DT)	Patient’s use of DT	Healthcare professional’ procedures in relation to DT
1	Early detection of pain in the joints (Ont i lederna)	If symptoms are detected by the evidence-based online-screening survey tool, it will recommend contact with primary care.	Data from the screening tool are not electronically shared with healthcare staff at the clinic. However, patients may bring a printout of their screening results.
2	Medical record online	Patients can access their medical record online anytime by logging in with electronic identification.	Healthcare staff know which data patients can access online.
3	Personal health plan online	A documentation about what was discussed during a clinical visit and what will happen next for the patient to feel safe and not forget the things she/he needs to remember. The plan is a complement to the medical record.	Healthcare staff write the health plan using terms their patients will understand. Decisions made during the meeting are documented (including both the patient’s and caregiver’s goals), including dates for the next meeting and lab tests, current medication and advice on self-management.
4	Online scheduling of meetings	Patients book their own appointments online.	Healthcare staff open up some of their appointment slots that patients can book. The others are only bookable through staff contact or referrals.
5	Patient’s own sample handling (PEP)	The patient can make self-referrals for lab tests and access their test results online, which allows them to monitor trends over time.	A nurse activates a specific lab test-package for each patient. Lab test results appear in the electronic health record and are processed the same way as ordinary lab tests.
6	Patient’s own registration of patient reported outcome measures (PER)	Patients answer questions about their health, e.g. level of pain and quality of life, before their visit to the RC. Patients can access their results online, and compare results over time.	Information from PER is used during the consultation for a joint discussion and comparison over time. The tool is connected to the national quality registry, and healthcare staff are regularly presented with aggregated PER data during staff meetings.
7	Online messaging	Patients log in to the national digital platform 1177 and securely send inquiries through a messaging function.	Nurses reply or distribute incoming inquiries. They encourage patients to use the messaging function instead of calling about questions or concerns.
8	Digital visit	The digital visit includes preparation using PER, lab tests (PEP) and filling in a digital form with questions and space for the patients to set a meeting agenda. Then the patients choose a physical or digital visit.	Healthcare staff prepare for the digital visit based on the information provided by the patient.
9	Care close to you – video meetings application	Patients can choose to have follow-up meetings through the video meetings application.	Healthcare staff offer digital follow-up meetings.
10	Application for self-management of physical activity (tRAppen)	Patients use the tool to track and self-manage their physical activity. The application connects patients with a group of “peers” with Rheumatic disease, thereby forming support groups.	Physiotherapists inform about tRAppen for those interested. Sometimes they offer telephone support to check if the patient has begun using it or needs some more help.
11	Elsa – a self-care application	A patient self-care tool for mapping life style habits and disease activity.	Healthcare staff can motivate patients to use the self-care application.

#### Strategy 1 – promoting early diagnosis and early contact with new patients

The RC offers an online screening service, *Ont-i-lederna (“pain in the joints”, #1),* that intends to promote early detection of rheumatic diseases. This screening tool is made available on the national web-based health service platform 1177.se. Some participants believed that the screening tool can motivate patients to seek care at an earlier time. Early diagnosis is important because it improves the prognosis. Individuals with a “positive” screening result are guided to book a consultation with primary care. A referral from primary care was necessary to get access to the RC.

As one participant explained, the healthcare staff at the RC do not have access to results from the online screening service. However, when the RC gets new patient referrals from primary care, they directly initiate a telephone contact with the patients. Participants emphasized the importance of this early telephone contact in order to make a timely assessment of the referred patient. The initial assessments helps the clinic to prioritize patients in urgent need of care, while the online screening service provide patients with an easily available opportunity to make an early assessment and initiate contact with primary care. Participants also emphasized that an early contact can be of psychological value for the referred patients, who may be calmed by knowing that they are not lost in-between healthcare providers or levels of care.

#### Strategy 2 – sharing of health information and health plans

Patients at the RC have access to their electronic health record through the online health platform 1177 (#2), which is not unique to the RC. Through this service, patients can access healthcare professionals’ notes from visits and other personal health information, such as lab results and prescriptions. The information shared through the electronic health record is not specifically tailored to patients’ needs, but the participants described that the RC offers an additional information sharing service that is more tailored to their patients’ needs. Patients at the RC have access to a *personal health plan (#3),* which is written in a language suitable for each individual patient. The personal health plan contains a patient-friendly documentation of the previous care visit and can also include self-care recommendations discussed.

#### Strategy 3 – offering digital patient-professional communication

Different services for digital patient-professional communication were identified. Participants described that the RC offers a service for text-based communication with their patients through an *Online messaging service* (#7), a digital service provided through the National Digital Healthcare guide (1177). While participants described an initial concern among healthcare professionals that they would be overwhelmed by messages from patients and not be able to manage to respond – this did not happen. On the contrary, it was even more convenient to exchange text messages with patients than being contacted by telephone. This was because asynchronous communication gave healthcare professionals more time to reflect and, if necessary, discuss patient inquiries with colleagues.

As an alternative to physical care visits, the RC also offers *Digital visits* (#8) through video consultation in a mobile application, “Care close to you”, developed by the region (#9). Prior to a digital visit, patients are required to fill in the web-based PROM form (#6) and an additional online form where they describe their problem and expectations for the visit. They also indicate particular needs, such as renewal of medical certificates or prescriptions. Finally, the patients may need to take a lab test prior to the visit, which they book through the patient’s sample handling service (#5). The participants explained that they suggest a physical visit if there are reasons for that based on the patient’s pre-visit information.

#### Strategy 4 – shifting tasks and initiatives from healthcare professionals to patients

The digital tools were used to enable patients to take over some of the tasks previously performed by healthcare staff. For example, an *online scheduling system* (#4) offered patients the opportunity to book appointments online when they needed to consult various healthcare professionals, which relieved healthcare staff from some administrative tasks. The participants described that this opportunity had led to a reduction in late cancellations and patients not showing up for their visits, in contrast to the appointments scheduled by healthcare staff.

Another tool*, Patients’ own sample handling* (#5), empowers patients to decide when and where to take blood tests for monitoring treatment response and/or disease activity, without a referral from healthcare professionals. We learned that this only works for certain predefined order sets, so-called “pre-visit tests” or “safety tests”. The pre-visit tests, as the name implies, are performed prior to a scheduled appointment. The “safety tests”, on the other hand, can be performed whenever patients perceive a change in symptoms and wish to follow up their disease activity. This service has offloaded nurses from administrative work related to sending lab test referrals to their patients at regular intervals. Participants believed that patients were motivated to use this service because they could access their lab test results online. When it was introduced, it was the only way to get online access to test results. However, in 2019 this had changed and access to test results online had been made possible also to patients that did not use the tool. Nevertheless, the participants also identified other potential motivators for patients to use the sample handling service. Specifically, they emphasized that patients are empowered to take more control and responsibility of their care. Some considered that this kind of service could be even more useful for patients with other diagnoses that may require more frequent monitoring.

Finally, the participants described a tool for *Digital collection of patient reported outcome measures (PROMs)* (#6) prior to each visit. Using a web-based form, also accessible at computer terminals in the RC waiting room, patients report for example experiences of pain caused by joint swelling and health-related quality of life. Based on the reported data, a disease activity score (DAS) is calculated and presented to healthcare professionals. The participants experienced that collecting PROMs prior to care visits, rather than during care visits, saved time that could be used to discuss issues that were important to the patient. Furthermore, they described that the structured and color-coded presentation of patient-reported data over time allowed them to see trends, which could serve as a decision support, in discussions with patients. The PROMs are also reported to the Swedish Rheumatology quality registry, which allows monitoring and comparison of quality indicators within and between clinics in Sweden.

#### Strategy 5 – providing support for self-care

The participants described that the RC offers different methods to provide educational disease- and treatment-related information to their patients. For example, they continuously update patient information on their website and provide printed brochures about areas of interest. The clinic also employs a (salaried) patient consultant to, among other tasks, provide consultation to patients about the different services offered at the RC.

The RC also offers different mobile applications to support patients in their self-care. One mobile application focuses on physical activity and peer support for individuals with rheumatoid arthritis – *Self-management of physical activity (tRAppen)* (#10). Patients who start using this mobile application get assigned to a group of around 10 users. The application enables patients to set personal goals and log their physical activity on their cell phone. Within each group, the patients can share experiences with each other and provide peer support. Another fairly new mobile application that focuses on lifestyle was described. This application *Elsa* (#11) enables patients to track different lifestyle and disease-related information. Healthcare staff do not have access to the data recorded in any of the two self-care apps, but the patient can use the information to discuss their situation.

### Strategies at the unit level for achieving PCC and improving chronic care

Five strategies at the unit level were identified, focusing on practices for quality improvement at the RC, in collaboration with patient representatives. The involvement of patients varied from indirect (e.g. routines to improve practices using registry data or other patient input) to direct involvement (e.g. patient representation in councils).

#### Strategy 6 – using regular follow-ups of quality indicators

The healthcare professionals described that they get quarterly reports at the unit level from the national Swedish Rheumatology Quality Registry. These reports on patients’ views and experiences are discussed during staff meetings to guide improvement work. However, the results presented can be difficult to interpret and somewhat misleading. As one of the participants explained: patients who feel worse are more likely than patients who are doing well to register PROMs on a regular basis. Therefore, the quality registry results (PROMs) may not fully reflect the overall clinical outcomes of the clinic’s patients. Participants shared the view that other sources of information are needed that in combination with the quality registry reports can guide their quality improvement work. In this strategy patients are indirectly involved.

#### Strategy 7 – arranging weekly unit meetings for continuous improvement and learning

Participants described that they experience a need to continuously learn and develop. This was partly facilitated through short weekly meetings in a multi-professional setting. Two different types of meetings were described. One type of meeting focused on rapid problem detection and idea generation. Healthcare staff gather around an improvement whiteboard where they discuss any identified problems and ideas for how they could be resolved and write down what is agreed on. As described, ideas need to be tested to find out what works and what does not work. The second type of meeting focus on ongoing research and development projects at the clinic. The staff gather weekly around a project whiteboard to inform and update each other about what is going on in the different projects, and document changes. A participant expressed that both types of meetings are educating and support planning.

#### Strategy 8 – operating as a test and improvement hub for digital tools

The participants described that the RC may be on the frontline of the development and adoption of digital tools for chronic care management in Sweden. One participant with a special responsibility for IT-related questions at the RC highlighted the need to work systematically with continuous evaluation and improvement of digital health technologies, and emphasized the importance of patient-involvement in development, testing and evaluation. To advance the development, the RC established a work group to define system requirements for the *digital visit service (#8)*. The RC has been the initiator or first adopter of several digital tools that have later spread to other settings and clinics, e.g. the online screening service [1], the patient’s own sample handling [5], the personal health plan [3], and patients’ own registration of PROMs [6]. It has therefore become apparent for the participants that the RC’s work on testing and initiating improvements of digital tools may also influence the healthcare digitalization movement in other areas of chronic care in Sweden.

#### Strategy 9 – collaborating with patient representatives in research and development

The RC has a *patient council* that consists of patient representatives interested in contributing to research and development. Participants had experienced that patient representatives with the courage to speak out their mind could be a great asset in the endeavor of achieving person-centered care. The contributions of the patient council were described to vary depending on identified needs and phases. For example, during the planning phase of the RC, the patient council met regularly to give input on the design suggestions for the new premises. Later on, the patient council provided input on the design of the clinic’s webpage or how to formulate referrals in a patient-friendly way.

The participants described that they also collaborated with the rheumatology patient association. Members of the patient association were invited to research and development meetings at the RC, as well as meetings with the strategic council at the academic specialist center. One of the participants suggested that it would be beneficial to also invite patient representatives to participate in the weekly improvement meetings at the unit. Besides the patients’ involvement in quality improvement at the RC, participants emphasized that a valuable aspect of the collaboration was that patient representatives have the power to advocate important issues in different settings, which may have an influence on both regional and national levels.

#### Strategy 10 – engaging patients in the waiting rooms in improvements

Besides the organized collaboration with patient representatives through the patient council or patient association, the participants described how also non-organized patients could be engaged in improvement initiatives. For example, an *“improvement box”* in the waiting room allows patients to contribute with ideas that are discussed in the weekly staff improvement meetings. Participants described that patient experiences were also collected through *surveys or individual interviews* conducted by the patient consultant.

Some participants had a feeling that good ideas or initiatives from patients were not captured well enough. A fairly new approach tested at the clinic was to dedicate some time for healthcare staff to circulate in the waiting rooms and ask patients about their opinions in so-called *“micro-meetings”*. One participant described how this could be a way to recruit patients to take part in specific improvement projects, for example to develop new educational material. Some participants were of the opinion that while the patient council was important during the establishment of the RC, the micro-meetings have become an even more important resource since the RC went into full operation. Nevertheless, they also experienced that this kind of spontaneous interaction could benefit from becoming a more structured and systematic work practice. Participants also expressed that the RC could be better at involving patients earlier in various development processes (see Table 3 for exemplifying citations of the results).

**Table 3:** Strategies and perceived impact of PCC practices and exemplifying citations

**Individual patient level**	
***Strategies***	***Citations***
1. Promoting early diagnosis and early contact with new patients	#1: Pain in the joints is more for primary healthcare. You can fill in the form before going to the primary care physician or nurse, and they will read it. When a (new) patient comes to us on referral, then this should already have been done. (Participant 2, round 2)
2. Sharing of health information and health plans	#3: At the end of the visit I print out the plan for the patient and we go through it together. “The goal is for you to be able to ski again, you shall contact the physiotherapist, you start with this drug and we follow up in 3 months. Lab tests will be taken and you will be contacted if we see anything, you can always contact us”. Then the patient knows what goes on and have it writing. (Participant 4, round 1)
3. Offering digital patient-professional communication	#8: You do your PER registration and lab tests as usual, but we have built a questionnaire, which was an idea that came from a patient who said,” I want to set my agenda for the meeting”, which is great. So we made a form and also included questions we tend to forget to ask, for example about dental certificates. Then you do not have to come here. Based on this form, the lab tests and the PER registration, you can choose whether you prefer contact via phone or video, or no contact if you feel well. (Participant 4, round 2)
4. Shifting tasks and initiatives from healthcare professionals to patients	#5: It is for the patient to gain power and knowledge and to become more involved. If we agree that the patient needs to submit lab tests once every 6 months, it is up to the patient to remember. I cannot force anyone to perform tests every 6 months and it is good to have less referrals so we can do more crucial things. So different reasons, but mainly for the patient to be able to do it on their own and access their results. (Participant 4, round 1) #6: I file pre-registered data on how the patient has felt in the medical record so that we can discuss what we think about it during the meeting. Patients become involved in how their disease has evolved over time and then they remember. (Participant 7, round 1)
5. Providing support for self-care	#10 & #11: We constantly try to work with these new mobile apps and try them out. We inform our patients about them so they can choose if they think any app could be valuable for them. This is how you have to work, to keep informing about what is available and then it is up to the patients to decide. (Participant 3, round 1)
**Unit level**	
***Strategies***	***Citations***
6. Using regular follow-ups of quality indicators	The quality registry send out reports every 3 months and we have started to use it a more actively during staff meetings. We will work with it to see what happens at this unit. (Participant 2, round 2)
7. Arranging weekly unit meetings for continuous improvement and learning	We have a whiteboard where anyone can post a note to be discussed during our meetings. It can be anything, positive comments, constructive criticism, something that works well, or experiences, a digital tool that is not working properly. It is very positive, it means that you are constantly questioning how we work, really think in new ways and try to develop what we do. (Participant 3, round 1)
8. Operating as a test and improve-ment hub for digital tools	The “Care close to you” application (#9) is being developed by the region and we participate as active as we can. With this app you will be able to do many things, case management, patients will be able to send images of rashes etc. We have high expectations on this application. (Participant 4, round 2)
9. Collaborating with patient representatives in research and development	Today’s presentation for the patient council of the app “Care close to you” is an introduction in order to create a project group that will work with the development of the app. Anyone who is interested is welcome to join the group. Everyone present at today’s meeting is informed that either they or colleagues who are particularly interested and/or knowledgeable are welcome. The project team will meet a few times during the fall and together work out desirable content for the app. (Document 10)
10. Engaging patients in waiting rooms in improvements	The formal patient council was important in the beginning and still is, but now we have more micro-meetings or direct conversations with patients. You dare to ask the patients directly what they thought about the visit, if there was something that didn’t feel good. (Participant 1, round 2)
**Perceived impact of strategies used for achieving PCC practices**	
***Perceived impact***	***Citations***
Shift in the patient role	E-health services help patients to involve themselves in their own care. With online access to their own medical record, their health plan with what we together talked about, and to beforehand send what you want to talk about we have opened up for talks on the things the patient finds important, not just what we want to know. (Participant 2, round 2)
Shift in the health care professional role	A patient who reads their medical record and lab tests and can check the adverse effects. It is good to share the responsibility, it makes me feel secure. To cooperate with the patient instead of putting all responsibility on me helps. A patient who contributes is what a physician needs. (Participant 9, round 2)
Behavioral and cultural change at the unit	Changes of attitudes and climate are needed, in both directions. Caregivers may not be used to including patients like this, and all patients may not be comfortable with being asked their opinion if they want the caregiver to know. So it is attitude and cultural change …not resistance. It does not always run entirely frictionless. (Participant 1, round 2)

### Perceived impact of the strategies used for achieving PCC

Three subcategories of the healthcare professionals’ perceptions of how work approaches and strategies had influenced patients and healthcare staff were identified*.*

#### Shift in the patient role

The introduction and use of digital tools was perceived to have enabled patients to get more involved in their care, resulting in several quality benefits. For example, participants perceived that information sharing enabled patients and healthcare professionals to communicate on more equal terms by reducing the asymmetry in information access. Services for enhancing flexibility in patient encounters were perceived to have improved patients’ access to healthcare and the view was that they thereby could contribute to a feeling of increased participation and safety among patients. By regularly making their own assessments about their health status and reporting PROMs through a digital service, the healthcare professionals experienced that patients had developed their knowledge and understanding about their own condition – which would enable them to take preventive actions.

Over time, patients were described to become increasingly independent in terms of self-care and therefore in need of more support tools to handle their condition and treatment. Participants emphasized that patients who want to take more responsibility should be provided with tools and opportunities to do so. While information-sharing from the direction of healthcare professionals to patients provides transparency and facilitates collaboration, some participants described how they had experienced that new channels for information-sharing could empower patients to participate more by becoming the initiator themselves. For example, the pre-visit form that patients fill in in connection with the digital visits (#8) enables patients to take responsibility in the preparation of care visits and care planning.

Participants described that digital tools, such as the patients’ own registration of PROMs (#6), contributed to patients’ learning about their condition and possibly how to prevent symptoms. They also acknowledged that not everyone was ready or suited for using the digital tools. Patients comfortable with yearly follow-up visits and telephone contact with the clinic may not be willing to take on more responsibility. It was emphasized that it is up to the patient to decide what services they are interested in and willing to use.

#### Shift in the healthcare professional role

Similar to the experienced shift in the patient role, participants described that the role of healthcare professionals also had shifted towards embracing collaboration with patients. One of the physicians acknowledged that they used to be bothered by patients who read a lot about their condition, but that their views had changed when understanding that it was easier to share decision-making with well-informed patients. Participants described how they had gained experience in educating patients to take more responsibility for their own health. For newly diagnosed patients, they had sometimes had to invest a lot of time in providing diagnosis and treatment-related information and self-management recommendations. Meanwhile, for patients who have lived with their chronic condition for many years and have good knowledge of their body-reactions and needs, it was more important for professionals to be good listeners in order to pick up the individual patients’ specific needs that can be further addressed. Participants also shared experiences of how their curiosity about the individual patients’ views had increased.

Contact with patients through text messages was another new task for nurses and physicians. Participants experienced that the easily available text messaging service for patients could have both benefits and challenges. One advantage was that neither patients nor healthcare professionals were bound to be available at a certain time. Participants described that they would often answer questions within a day or so. A challenge was that the messaging service could occasionally be heavily used by patients at times when they were particularly anxious.

Some challenges were experienced by the healthcare professionals when adjusting to new work approaches. One challenge was the frustration with the e-service platform 1177, which was experienced as technically inert. Also, they perceived that technical development was slow. Meanwhile, or maybe because of that, they also experienced difficulties in adapting their own work practices to using digital technologies in collaboration with their patients. Several of the available digital tools remained underused.

Further, one physician described fewer physical visits per patient, which saved time and enabled physicians to see more patients. However, the clinical work had thereby become more challenging because more patients implied a greater medical responsibility. The increase in remote contact with patients using the online messaging and video meeting services for routine care also implied some challenges. Nurses take the main responsibility for digital communication with patients, and had also taken over some of the routine follow-up visits previously performed by physicians. One of the participants recognized that while simple or routine questions can be managed through digital communication or task shifting, the physical visits are increasingly characterized by more challenging medical questions. However, the participants experienced that new ways of digital communication and collaboration had not jeopardized safety even if physical visits were less frequent, and that they continuously worked with developing routines for assessing which type of meetings that was most suitable based on the patient’s needs and preferences.

#### A behavioral and cultural change at the unit

The interviewed healthcare professionals were in agreement that they strive towards a situation where patients and healthcare professionals work in partnership, contributing with their respective expertise and experiences to achieve better individual health and a good work environment. However, they also acknowledged that formal patient participation in quality improvement at the unit is still in its early stages. Participants expressed that it was challenging to change their previous ways of working, especially for healthcare staff with many years of working experience. Similarly, some experienced that this type of cultural change could be challenging also for patients who have many years of experience with their chronic condition and of being a patient in the traditional healthcare system. The participants described that when they started to involve patients in discussing the clinic’s operations, it was still quite unusual for patient associations or individual patients to have a say in these type of matters. Patients did not know what to expect. We learned that healthcare staff had gone through a process of establishing relations and building trust, so that patients would understand that the RC was serious about working together to improve the quality of care. Participants experienced that the atmosphere of inclusion of individuals and ideas, and the readiness to test new things, distinguished the RC from other workplaces.

 Participants also revealed that not all patients and healthcare staff were equally enthusiastic. The continuous small-scale testing of new work approaches and digital tools required behavioral changes of both patients and healthcare staff. Healthcare professionals were described as not being used to include patients in their work routines, and patients were not used to being involved. Some of the participants described that it was particularly challenging to be transparent about experienced shortcomings. For example, when patient representatives were invited to staff meetings, it was experienced as more difficult for healthcare staff to express their skepticism about, for example, some of the digital tools and services that may be appreciated by patients.

## Discussion

This study aimed to investigate the overall strategies to achieve PCC used by the healthcare professionals at an outpatient rheumatology clinic, the strategies’ relation to digital tools, and the perceived impact of the strategies on healthcare staff and patients. Ten strategies were identified, five at individual patient level and five at the unit level. The strategies involved new practices related to digital tools, which were perceived as useful new ways of getting patients involved in their own care, affecting both the healthcare professional and the patient role. The study provides an empirical example of the process of achieving PCC in chronic care and the role of digital tools in this process, from the perspective of healthcare providers.

### Strategies for achieving PCC in chronic care

The five strategies for achieving PCC at individual patient level described practices when interacting with individual patients to improve treatment and care of their chronic condition, and how digital tools were used in this process. At the unit level, the collaboration with patients concerned improvement of routines, services and digital tools offered at the clinic. At both levels, the strategies broadly concerned accessibility and adaptation to individual needs.

The individual level was evident in this study and the digital tools at the RC were designed and used for enabling person-centered interactions and communications before, between and during care meetings with patients. The PCC strategies at the individual level concerned both medical aspects of monitoring the disease to personalize the treatment and social aspects of the interaction between professional and patient and the patient’s situation to identify important needs and personalize communication and care approaches. The strategies also span over a continuum of engagement, from consultation to partnership and shared leadership [23], where strategy 4 and 5 refers to the higher end of the engagement continuum.

While many studies and descriptions of PCC focus on the individual level, i.e., the interaction between caregivers and patients [43], Santana and colleagues [15] emphasize the structural conditions on the system and organizational levels in order to provide the foundations for PCC on provider-patient level. We also identified supporting organizational strategies for achieving PCC practices. Such strategies can manifest themselves in organizational culture, structures, procedures, and professional development practices [43]. Changes are required on the organization and system levels in order to support and maintain PCC processes and outcomes [15]. To achieve PCC in healthcare cannot solely be up to individual health professionals without support from the organizational or system levels [33].

At the RC, some patients were involved in unit level improvements, usually designed with the aim to utilize and capture patients’ opinions and needs. Patients were involved directly in two ways: as patient representatives in the unit’s patient council and in various meetings or in specific situations when asked to participate. Patients had participated in the planning of the layout of the RC’s new premises, in the development of new digital tools and in improvements of care practices at the RC (e.g., a preparation form used prior to digital visits, #8). Patients had also been more indirectly involved in improvement of care practices and other routines by systematically providing their opinions and experiences. For example, by reporting PROMs that the national quality registry for Rheumatology compiled and presented on national, regional and unit levels, which the clinic regularly used to aid improvement.

However, while patients were encouraged to use, evaluate and provide feedback to improve the digital tools, they were more seldom part of initiating or designing new services, tools or work practices. Patients were more reactive to healthcare staff’s ideas than proactively providing their own ideas or solutions. The interviews indicated that the healthcare professionals had recognized this gap and strived to include patients in all parts of the development process, while also acknowledging that development of new digital tools and practices takes time, as they involve technical development and changes in habits and work practices. If the view of the patient as a partner persists and is practiced for some time, then the participants’ perceived that this could change. The process of becoming an innovative unit and test-hub for digital tools and PCC in Rheumatology care had started before the new RC organization was launched in the new premises [44]. During the innovation processes even more attention may need to be payed to the dimensions of interaction, also at the system level, in order to partner with patients to comprehensively develop digital tools to aid PCC.

### The role of digital tools in PCC practices and improvement of chronic care

Several digital tools were used for collaboration with individual patients. The tools had different purposes: early diagnosis, sharing of health information, task shifting, digital communication and support for self-care. In that sense, the digital tools promoted different components of PCC. Some functions can be regarded as basic preconditions for collaboration, such as sharing of information, while others are more sophisticated, such as handling your own lab samples (#5) or planning for your visit (#8). Carman et al. [23] has described these dimensions as a continuum of engagement from mere consultation, via more involvement to a genuine partnership and shared leadership, with the aim to move towards the higher end of the continuum. The digital tools at the RC have in common that they shift some responsibility to patients, which requires a learning process. To what extent they are used is dependent on motivation and the acquired knowledge and experience over time during this process. It has been suggested that the development towards PCC can be advanced by the use of digital tools to increase patients’ beliefs in their own capacities to manage their own care [29]. Our analysis revealed that the digital tools were perceived to enable mutual sharing of information, increase transparency and allow for more flexible contacts with patients. The digital tools were used for medical aspects of monitoring and treating the chronic condition for each patient, for social interaction and for increasing mutual knowledge. Thus, the digital tools can be regarded as enablers of PCC by empowering patients through providing easier access to information and increasing their knowledge about their chronic condition [15]. A core component of PCC is that patients can feel an ownership of their condition, and ICT can aid both patients and providers in the PCC process [45]. This was accomplished by providing access to medical records, lab test results and self-reported outcome measures, as well as by patients informing the care provider and setting the agenda for meetings through the digital visit form. Tools were also used to make care more accessible by the use of online time booking, video-meetings and email-communication. This enabled a more flexible communication for both patients and healthcare staff. In that sense, the digital tools made collaboration possible on more equal terms. The results also indicated that the tools should be regarded as enhancing PCC rather than replacing other care practices.

Digital tools per se do not enhance PCC practices unless they for example aid patients to know more about their condition, facilitate patients’ involvement in their own care, or promote communication about things that matter for them [46]. Given this, PCC practices can increase the consensus between patient and caregiver concerning what should be done in a certain situation for a certain patient, which in turn can increase the motivation to execute what was agreed [47]. The RC invites patients to become more involved in their care by using the provided health tools, but how far the practicing of these tools will reach depends on if patients find them useful. At the RC, focus has moved from developing separate digital health tools with a specific function towards creating a tool-box where several digital tools are gathered. Then healthcare professionals and patients can choose tools according to each specific situation and the specific patient’s needs. Only a few digital tools in the tool box focused on self-care, while some had preventive functions and gathered data to enhance learning over time. The existing tool-box may need to be complemented with new solutions and initiatives for prevention and health promotion – that can include other types of actors, external or internal to the healthcare organization.

### The impact of strategies for achieving PCC and new digital tools for patients

The RC was initiated to manage rheumatic conditions according to patients’ needs in a more sufficient way. Changes in work practices when achieving PCC implies a process of changing behavior and roles for both healthcare professionals and patients. This has been described as going from paternalism to partnership [48]. In order to work towards such a development, certain questions need to be raised. For example about roles and responsibilities and the organizational conditions required. The participants described a shift in the patient-role from being a care-receiver to becoming more independent by using digital tools and thereby allowing patients to act more as a team player. However, a one size fits all solution was not considered appropriate. Instead healthcare professionals needed to be attentive about when and what to introduce and inform about depending on the individual patient. PCC practices imply being focused on the individual patient’s situation and needs, and in that sense being able to move from traditional to new ways of working [49]. These new ways of working for healthcare professionals have also been described as taking on a more coaching and supportive role [50]. Healthcare professionals need to handle patients that can be in different states of independency and able to take on more or less responsibility for their care. The professional role was described as having the patient as a team player in care situations, and coaching patients to take greater responsibility for their own health was considered the path forward.

Some of the healthcare professional’s work tasks were reduced and/or replaced by others, e.g., fewer physical appointments and more digital communication, and a new type of preparation before meetings to free time for dialogue about what matters to the patient. We identified both a shift in the healthcare professional’s formal role (formal responsibilities, tasks etc.) and a shift in what was expected from the RC unit in terms of being an innovative test hub, enabled by a special reimbursement model. Changes also involve healthcare professionals’ softer skills (e.g., social interaction, communication style) and social aspect of their role (e.g., role expectations not formally described) [51, 52]. Empowering patients via new tools and opportunities to act also implies that changes in healthcare professionals’ behaviors to allow this shift more or less have to take place. Some of the descriptions in the interviews highlighted that such changes were needed while others that some had taken place. However, the informants provided few examples of these softer aspects of behavioral change and situations where patients are prepared to take part, know what is expected from them and can speak their minds.

Achieving PCC practices is also connected to a change in organizational culture [53], in this case creating a PCC culture [15]. Such change was only indicated and/or seen as important by some informants, but not possible to firmly establish in our study. This would have required an extended timeframe and complementing data. Further, WHO suggest that PCC, or people centered practices, include an integration of health services between healthcare providers and other actors relevant to the specific patient [54]. This aspect was not mentioned by the participants and depending on the organization of health care it is beyond the scope of a specialist unit. Nevertheless, integrated care is an important issue to consider, especially for patients with multiple conditions.

### Study limitations

This study is mainly explorative and contains two complementing data sources – interviews and documents – collected at two different time points. The semi-structured interviews provided in-depth data and participants were able to reflect on the questions and their own experiences and views. The documents provided information on the unit level, validating and complementing findings from interviews. The feedback session at the RC, where all healthcare staff were invited, served two purposes: to validate the results from the first round of interviews, and to identify any gaps or changes to follow up on.

The study had a provider perspective on PCC and involved only health care professionals that were identified as having experiences of using digital tools for PCC and healthcare professionals working more than 50% off full time at the unit. As the unit had many part time employees it is possible that some findings would have been more or less prominent if all of them had been interviewed. Further, the study is limited to one outpatient clinic and findings are primarily transferable to similar chronic care settings with similar missions. Patients’ experiences should also be explored in order reflect their views on work practices and challenges described by the healthcare professionals. A forthcoming study will describe the patients’ perspectives on PCC practices and the use of digital tools at the RC. Further studies of PCC practices and the role of digital tools can also benefit from an extended sample where questionnaires could provide information from additional clinics, and if possible observations of work practices.

## Conclusions

This study provides an empirical example of the strategies used to achieve PCC in a chronic care clinic and the role of digital tools developed for patient use in this process, from the perspective of healthcare providers. The study contributes to the knowledge on PCC practices by expanding our understanding of how digital tools and work practices interact and how they may affect healthcare professionals and patients during the process of achieving PCC in chronic care, on the individual patient level and the unit level.

The main conclusion is that the use of various digital tools, spanning over different dimensions of required engagement, facilitated the interaction with patients and their involvement in their own care, from the perspective of healthcare providers. The digital tools served as a toolbox with different purposes – and supported various aspects of PCC, but less so for prevention and self-care. The digital tools did not replace other care practices, rather, they served as a complement for patients that were able to use these tools. Tasks and roles shifted due to the introduction of the digital tools, both for health care professionals and patients, something that needs consideration when implementing PCC and digital tools to support PCC. The ten strategies identified can aid other outpatient chronic care clinics. Hopefully they can learn from these experiences and adopt similar or further developed strategies and digital tools. Even so, there will always be a need to consider the specific organizational context (enabling PCC practices) and the specific social context (healthcare staff, management, patient population and individual patients).

Our study highlighted some questions on how to further develop the process to achieve PCC. Especially the support and tools for prevention and self-care provided, the ways to increase the patients’ involvement in the development of valuable and user friendly new digital services, and ways to support individual patient’s and health care professional’s ability to use them.

## Supplementary Information

**Additional file 1.**

## Data Availability

The qualitative data for this study is safely stored by the department of Learning, Informatics, Management and Ethics at Karolinska Institutet in Sweden. It consists of transcripts from semi-structured interviews and documents retrieved from the participating organization (all text in Swedish). It is available on request to the first author and after signing appropriate documents in line with the ethical application and Ethics Board’s decision. Excerpts from the data sources are presented in the article as citations (translated into English).

## Abbreviations

RC: Rheumatology clinic
PCC: Patient and person centered care
ICT: Information and communication technology
Healthcare staff: All staff at the unit
Health care professionals: Those professions included in our sample i.e. nurses, physicians, physiotherapists, and occupational therapists
