# Supplementary material for: Digital tools as promoters for person-centered care practices in chronic care? Healthcare professionals’ experiences from rheumatology care
Source: BMC Health Serv Res. 2020 Dec 1;20:1108. doi: 10.1186/s12913-020-05945-5 (PMC7709268; doi:10.1186/s12913-020-05945-5)
Supplement: Supplementary file 1 — Additional file 1. [file 12913_2020_5945_MOESM1_ESM.zip › Interview guide first roundR3.pdf]

Interview guide – First round

A) Background

1) Can you briefly describe your professional role and function/responsibilities at the Rheumatology clinic (RC)?
2) How long have you been working at the RC?
3) What is your experience of working with quality registries in healthcare? What is your general view on the use of quality registries in healthcare?
4) What is your experience of working with e-health services in healthcare? What is your general view on working with e-health services in healthcare?

B) Organisation

The RC and its mission
In the RC's formal mission it is written that the RC is an "academic unit" - where care is integrated with research, development, education and innovation (R&D).

5) What are your expectations of the RC regarding these parts of the assignment? As parts, and all together?
6) Has any part of the assignment (*care integrated with research, development, education and innovation)*, so far, been given a special priority in your daily work, apart from care?
7) Can you give examples of how you have worked with research, development, education and innovation?
8) Have you encountered any challenges in this work? If you have, please describe.

The mission also includes "developing a) new ways of working, b) innovative use of medical technology and e-health solutions, c) high patient participation/involvement, and d) collaboration with other healthcare providers ".

9) What are your expectations of the RC regarding these parts of the assignment (a-d)?
10) Has any of these parts of the mission (a-d), so far, been given special priority in your daily work?
11) Can you give examples of how you worked with a-d?
12) Have you encountered any challenges in your work with a-d? Please describe.
13) How do you work with patient participation/involvement?
    a. What is the role of patients in the work you describe?
    b. How does it differ from your previous experiences and from potential previous workplaces?

Context and the establishment of the RC with its new mission

14) What circumstances have facilitated the establishment and development of the new RC?
15) What circumstances have made it difficult to establish and develop the new RC?
16) Which, if any, measures have been taken to deal with the above mentioned difficulties?

Results

17) What results have you already seen, linked to the RC's goals of developing:
    a. new ways of working?
    b. innovative use of medical technology and e-health solutions?
    c. patient participation/involvement?
    d. collaboration with other healthcare providers?

Continued establishment and development

18) How can the RC's operations and services be further developed towards the mission's objectives in a-d?
    a. In the short term and long term perspective?

19) What is needed for this development in the areas a-d to happen?
20) Which actors do you think can play an important role in this development?
21) Do you have any additional information about the RC and a-d that you want to mention?

C) The use of e-health services and digital tools at the RC

1) Which e-health services/digital tools used at the unit are you involved in/do you use?
2) Can you describe on a more general level why you work with these e-health services/digital tools? *(Relation with a-d?)*
3) What are you trying to achieve by using several different e-health services/digital tools? *(Relation with a-d?)*

Questions linked to each one of the e-health services/digital tools the respondent uses:

Use and goals

4) What are you trying to achieve with the e-health service/digital tool? What is the intention/purpose?
5) What professions/occupational roles uses the e-health service/digital tool?
6) How do you practically work with the e-health service/digital tool?
7) How do you interact with the patient in relation to this e-health service/digital tool?
8) What part of the patient group do you most often use the e-health service/digital tool with? Why?
9) At what time point of the disease process do you use the e-health service/digital tool? Recurrently or just once?
10) What do you do in your role to support the use of the e-health service/digital tool? Directed to patients, at other staff, and/or at management levels?

Results and effects

11) In your opinion, how do you think the work with the e-health service/digital tool at the RC is functioning?
12) What does the e-health service/digital tool contribute to? *(Relation with a-d?)*
13) What are the effects of the use of the e-health service/digital tool? *(Relation with a-d?)*
14) Have you experienced any unexpected effects of the use of the e-health service/digital tool?

Setting and process

15) What is needed to fully use e-health services/digital tools in line with their intentions? *(Relation with a-d?)*
16) What has facilitated the use of the e-health services/digital tools at the RC?
17) What has made the use of the e-health services/digital tools more difficult at the RC?
18) Do you have any suggestions for development in relation to the e-health services/digital tools?
19) Are there any forums for you to address the staff's views and experiences in using e-health services/digital tools? Which ones? What do they address? Who is involved?
20) Is there a forum for addressing patients' views and experiences in using e-health services/digital tools? Which ones? What do they address? Who is involved?

21) How does using multiple e-health services/digital tools work in combination?
22) What is the role of e-health services/digital tools in improving and developing healthcare and RC's work (a-d)?

Finishing question

23) Is there anything more you would like to add?
