# Supplementary material for: Digital tools as promoters for person-centered care practices in chronic care? Healthcare professionals’ experiences from rheumatology care
Source: BMC Health Serv Res. 2020 Dec 1;20:1108. doi: 10.1186/s12913-020-05945-5 (PMC7709268; doi:10.1186/s12913-020-05945-5)
Supplement: Supplementary file 1 — Additional file 1. [file 12913_2020_5945_MOESM1_ESM.zip › Interview guide follow-up interviewsR3.pdf]

Interview guide – Follow-up interviews

A. Approach to patient involvement and patient participation

1) Can you describe how you work to promote patient involvement and participation at the Rheumatology clinic (RC) today?
2) Any changes since the last interview?

B. E-health services/digital tools - use, results and effects

In the last interview we talked about the e-health services/digital tools used at the RC.

3) Do you still offer the same e-health services/digital tools or have some disappeared or new ones been introduced?
4) If there have been changes - Why? (Changes in priorities? Changes in patients' use/demand/needs? Other?)
5) Have there been any changes in the purpose or goal of using these e-health services/digital tools?
6) Have there been any changes in their practical use?
7) Have there been any changes in how staff and patients interact in relation to the e-health services/digital tools?

Results and effects

8) So far, what are the results and effects of using these e-health services/digital tools?
9) What do e-health services/digital tools contribute to and how do they affect:
    a. The meeting between patient and healthcare staff?
    b. Staff's work approaches and/or development of work approaches (individual/within/between professions)?
    c. Development of the RC (e.g. overall routines, organization, division of work)?
    d. Development outside the unit (e.g. dissemination to other units within the organization/other rheumatology units)?
    e. Anything else they affect?

C. Procedures for patient participation/involvement and development work at individual patient and unit levels

Individual patient level

10) To what extent would you say that patients are involved in their own care at the RC at present?
11) In what parts of the care process is the patient involved - in assessing, in planning and setting goals, in performing agreed treatment, in evaluation? Variation?
12) Can you give examples of how you work with participation/involvement in these parts of the care process?
13) What you have described, is this way of working established in routines or in practice at the RC or is it more of a free choice for each person if they want to work that way?

Unit level

14) To what extent would you say that patients are involved in developing the services and the way of working at the RC?
15) Are the patients involved in any specific occasions or development areas?
16) Can you give examples of how you work with patient participation/involvement on these occasions?
17) What you have described, is this an established routine or practice or is it more a free choice if you want to work that way?

Individual and unit levels

18) What has facilitated your work with patient participation/involvement so far at both individual patient and unit level?
19) What has made it more difficult for you to work with patient participation/involvement so far at both micro and unit levels?
20) Has your work on patient involvement/participation left any ideas on how to organize the work at the RC at a more general level?
21) Have you experienced any unexpected positive or negative reactions or effects of the RC's approach to promoting patient participation/involvement, with or without e-health services/digital tools?

D. Information/data collected in relation to development

22) Do you use information collected in the SRQ national quality register for development? If yes
    a. How do you go about using the information for the development of the care for individual patients?
    b. How do you go about using the information for developing the working method at the RC?
23) Are there any other sources of information or e-health services/digital tools used for device development? If so, which ones?
    c. How do you go about using these sources of information for the development of care for individual patients?
    d. How do you go about using these sources of information for developing the way of working at the RC?
24) What has made it easier for you to use different sources of information for development at the individual patient and unit levels?

25) What has made it more difficult for you to use different sources of information for development at the individual patient and unit levels?

E. Finishing question

26) Is there anything more you want to add to help us understand your experience and how you work with patient participation /involvement; e-health services/digital tools, and development at individual patient care and unit levels?
